# Superior Longitudinal Fasciculus: A Review of the Anatomical Descriptions With Functional Correlates

**DOI:** 10.3389/fneur.2022.794618

**Published:** 2022-04-27

**Authors:** Felix Janelle, Christian Iorio-Morin, Sabrina D'amour, David Fortin

**Affiliations:** Division of Neurosurgery, Department of Surgery, Faculty of Medicine and Health Sciences, Université de Sherbrooke, Sherbrooke, QC, Canada

**Keywords:** superior longitudinal fascicle (SLF), association fibers, white matter tracts, diffusion imaging, MRI

## Abstract

The superior longitudinal fasciculus (SLF) is part of the longitudinal association fiber system, which lays connections between the frontal lobe and other areas of the ipsilateral hemisphere. As a dominant association fiber bundle, it should correspond to a well-defined structure with a clear anatomical definition. However, this is not the case, and a lot of confusion and overlap surrounds this entity. In this review/opinion study, we survey relevant current literature on the topic and try to clarify the definition of SLF in each hemisphere. After a comparison of postmortem dissections and data obtained from diffusion MRI studies, we discuss the specifics of this bundle regarding its anatomical landmarks, differences in lateralization, as well as individual variability. We also discuss the confusion regarding the arcuate fasciculus in relation to the SLF. Finally, we recommend a nomenclature based on the findings exposed in this review and finalize with a discussion on relevant functional correlates of the structure.

## Introduction

Although brain surgery for intrinsic glial tumor has greatly evolved in the last decades, progress in this discipline has been hampered by incomplete knowledge of the brain functional anatomy (1). One of the main challenges of glioma brain tumor surgery is to follow the principle of maximal safe resection, which is to remove as much of the tumor as possible while preserving the healthy surrounding tissue to minimize functional loss and preserve the quality of life (2). This is especially challenging for infiltrative or diffuse tumors such as gliomas, which present no clear boundaries between tumor and normal brain parenchyma. To achieve this goal, it is paramount to protect the integrity of relevant cortices as well as intact peritumoral white matter bundles. As such, a pursuit in improving the anatomical and functional knowledge of cortical and subcortical structures is in keeping with this objective.

The superior longitudinal fasciculus (SLF) is considered to be the largest associative fiber bundle system in the brain. The SLF is a part of the longitudinal association fiber system, which lays connections between the frontal lobe and other areas of the ipsilateral hemisphere. To put it simply, it connects the perisylvian areas in the hemisphere (frontal, temporal, and parietal). As such, this fiber bundle is likely to be one of the most affected whenever we undertake a surgery for an intrinsic brain tumor. Although our interest in refining the definition of the SLF stems from our work in infiltrative glial brain tumors, a clearer definition would also benefit all spheres of clinical neurosciences. Hence, the goal of this study is to review SLF anatomy, nomenclature, and function. Interestingly, one would expect that this fiber bundle would already be thoroughly portrayed and delineated by now, and that this modelization would meet with a large consensus; as we will see, nothing could be further from the truth!

### A Brief History of the Birth and Characterization of the SLF Anatomy

Reil and Autenrieth, pioneers of connectional anatomy, identified the SLF using postmortem brain dissections at the beginning of the 19th century. The first description coined it as a group of fibers located in the white matter of the temporal, parietal, and frontal lobes (3). This initial description was further refined by Burdach, a contemporary of Autenrieth, followed by Dejerine in 1895 (3, 4). These authors unveiled a peri-Sylvian arch-shaped fiber tract connecting the posterior temporal lobe with the frontal lobe. They named this bundle the arcuate fasciculus (AF) because of its shape and used the term “superior longitudinal fasciculus” as a synonym, introducing a confusion that still persists today (3, 5). A century later, studying the rhesus monkey by means of autoradiographic technique, Petrides and Pandya divided the SLF into three distinct segments (6, 7). These authors distinguished the SLF and AF as two distinct entities with different pathways, blurring the classical description prevailing at the time (6).

Until the early 1990s and the advent of diffusion magnetic resonance imaging (MRI), the anatomy of the SLF was studied in non-human primates using axonal tracing, a technique considered the “gold standard” in unveiling connectional anatomy of white matter *in vivo* (7, 8). Hence, this led to a paucity in human-derived data for this period (7–10). The arrival of diffusion imaging and fiber tractography changed all that, allowing the study of the human brain connection modelization *in vivo* (11–13). The use of this technology has helped elucidate some controversies regarding the SLF anatomy data derived from postmortem dissection (14, 15).

### Anatomical Description Derived From Postmortem Dissections

Martino et al. (16) developed a modification to the classical fiber dissection methodology, initially designed by Klinger (15). The idea was to preserve the cortex by removing minimal tissue during dissection, hence producing a cortex-sparing fiber dissection. In the first step of the cortex-sparing fiber dissection, a wooden spatula is used to remove the cortex within the depth of the sulci only, preserving the cortex of the convexity surface of the gyri. This is crucial as it skeletonizes the stems of the gyri, providing space to dissect the white matter while preserving the cortical anatomical landmarks. This allowed the study of the fiber trajectory and the orientation within the white matter, as well as an estimate of cortical anatomical connectivity that diffusion imaging is still unable to produce. Using this approach, two superficial segments of the SLF were identified: the first, which is horizontally oriented connects the inferior parietal lobe and the posterior portion of the superior temporal gyrus with the frontal operculum. The second component runs along the AF and connects the posterior portion of the middle temporal gyrus with the posterior portion of the inferior parietal lobe (the angular gyrus). A deeper fiber segment that corresponds to the classical AF was also identified (3).

However, not all investigators found evidence of the presence of SLF during their dissection. Studying 10 consecutive cadaveric brains in search of long horizontal fronto-parietal association bundle in the white matter, Maldonado et al. (17) could not identify the SLF after having removed the short “U fibers”. This illustrates the limitations of postmortem dissection, a technique fraught by constraints adversely impacting its validity (7). Indeed, this macroscopic dissection can not only resolve fibers crossing or follow fibers for a long distance but is also unable to identify the distal terminations of bundles (3, 7). As the brain commonly used are from elderly subjects, the technique is inherently biased toward old age, and potential effects of exposure to pharmacological treatment, nutritional status, medical condition, and cause of death also represent other potential biases. Postmortem factors, such as the interval between death and fixation, as well as the known effects of the fixative on the tissue can also alter results (18–21). Hence, the necessity to use an *in vivo* method to carry these studies (22, 23). Moreover, it obviously is a macroscopic approach for the resolution of a microscopic architecture!

### Anatomical Description Derived From Diffusion MRI Studies

MRI is based on the fact that hydrogen atoms in water molecules act like protons having a spin that can respond to a magnetic field or gradient. Protons align to this external field, and it is possible to assess their spin and their decay back to their relaxed state after having been excited by an RF pulse (24). Investigators are constantly pushing the boundaries of the technique to design new imaging sequences and applications.

#### What Is Diffusion MRI?

**Figure 1:**
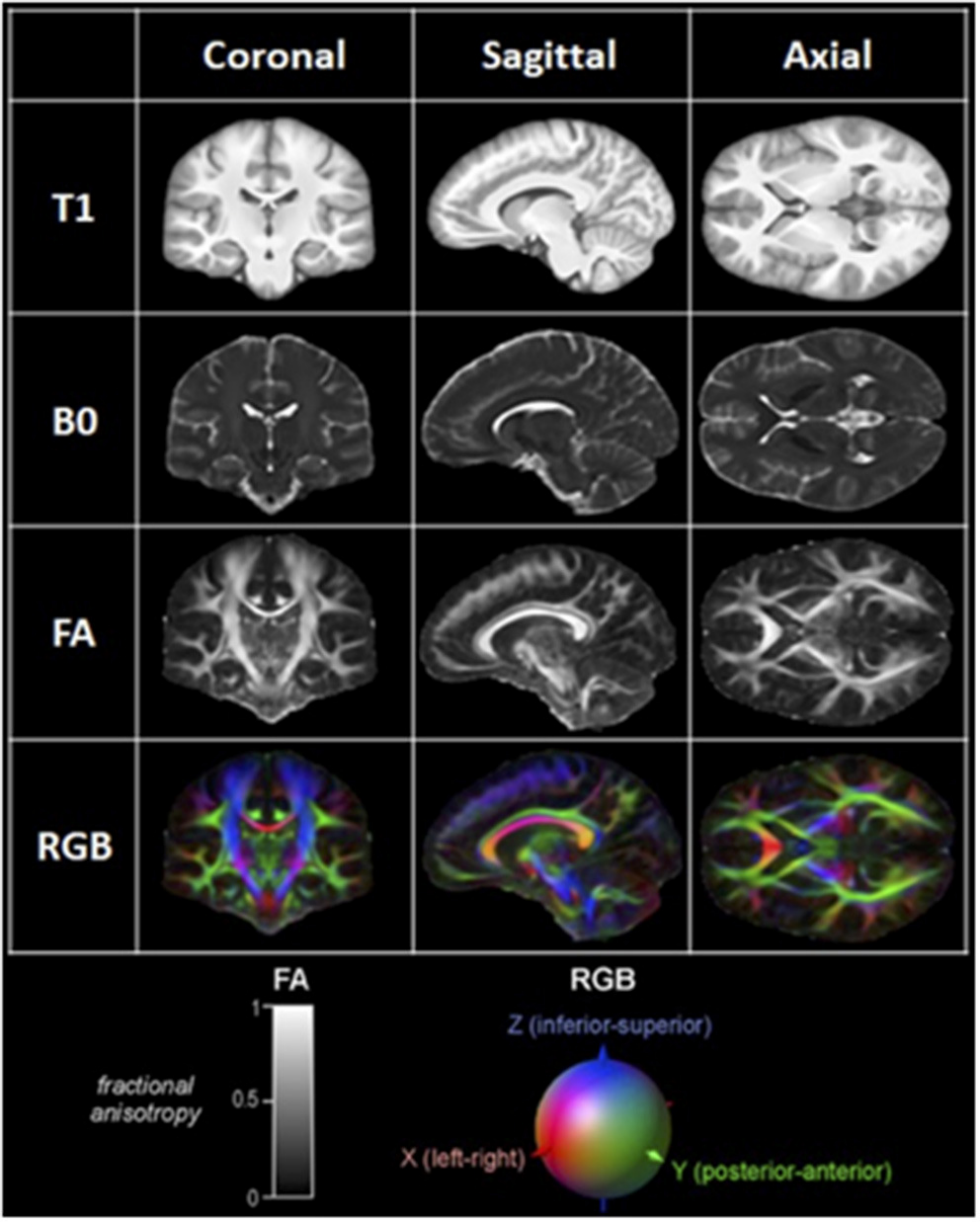
Examples of coronal, sagittal, and axial MRI and DTI images. T1: T1-weighted image. B0: B0 image. FA: Fractionnal anisotropy image; Representation of the level of anisotropy in each voxel on a scale of 0 to 1, as shown in the lower left corner. RGB: Red-Green-Blue Color-coded FA image; Color-coded representation of the main direction of diffusion in each voxel, as shown in the lower right corner.

Diffusion MRI is an imaging modality that scrutinizes the diffusion of water in the brain. In a free medium, water molecules normally display Brownian motion, i.e., they diffuse equally in all directions. When these molecules are in and around axons, however, their movement is hindered and restricted by axonal and dendritic membranes, glial cells, and myelin sheaths (25). As a result, their net diffusion is higher in the direction parallel to the fibers. This directional diffusion can be surveyed by the MRI scanner by applying magnetic gradients and taking measurements from different directions. The more structured and organized the axonal tissue is within a voxel, the more it is said to be anisotropic, and the more likely there is a white matter bundle going through this voxel in a singular direction (26–29). Diffusion MRI is classically modeled by a diffusion tensor, which is a single-fiber model per voxel projecting an estimate of the principal direction of diffusion in 3D. This model is at the heart of diffusion tensor imaging (DTI) (28). Classically, fractional anisotropy (FA) map or red–green–blue (RGB) colored directional images are used to represent diffusion data (24, 30) (Figure 1). Once the data is acquired, it is processed using mathematical deterministic or probabilistic algorithms to “connect” coherent diffusion tensors from adjacent voxels to produce a simulation of tracts; this process is called tractography.

Since the inception of DTI, newer techniques have emerged (advanced dMRI), refining both the scanning sequences, as well as the tractography algorithms (31, 32). Indeed, increasing the number of directions measured per voxel allowed the creation of high angular resolution diffusion imaging (HARDI), in which the resolution of multiple fiber populations crossing within the same voxel is now possible (33). Other such next-generation of refined techniques are diffusion spectrum imaging (DSI) (34–36), diffusion kurtosis imaging, (37–39) and q-ball imaging (40, 41). These newer approaches were designed to solve the problems generated by cases where fibers are crossing, kissing, fanning, or bending within a singular voxel (42). This is a problem of considerable importance since it is estimated that 63–90% of white matter volume contains crossing fibers (43).

#### Is DMRI Reliable in Revealing White Matter Anatomical Structures?

To this complex question, we can now safely answer yes, but with a few distinctions. Classical DTI postmortem histological validation studies have shown that fiber orientation is correctly represented in large unidirectional fiber bundles, but fails in complex regions with fiber crossings and low anisotropy (44–46). Newer studies using refinements to dMRI mentioned earlier are promising in solving these complex issues (47).

Fernandez-Mirannda et al. (48) mapped the whole brain using advanced dMRI and validated the tractography findings by dissecting 20 normal brains. Their results showed that advanced dMRI overcame DTI challenges in multiple areas such as the cortical and subcortical termination of fibers, decussation of fibers, zones of triple crossings, high and complex angulations, terminal arborization of fascicules, and cortical connectivity. Various investigators have studied specific white matter bundles with dMRI by comparing and validating results with the anatomy observed by postmortem microdissection (7, 47). Overall, the results were in support of an adequate validity and reproducibility of the technique with some nuances. However, there is one domain where histological postmortem studies have shown a consistent failure of dMRI, which is in regions of transition between white and gray matter, where orientation errors of as much as 90° have been observed (49, 50).

Hence, this imaging technique is greatly evolving and shows great promise (51). However, certain methodological pitfalls remain, and validation with other measures should be encouraged, as the technique is not entirely mature yet. The essence is to distinguish the extent to which these methodological shortcomings impact the validity of the tractography results. As a warning, Maier-Hein et al. (52) reported that this impact could be considerable. Indeed, these authors organized an international tractography competition and drew several striking conclusions. One of them was that current state-of-the-art tractography algorithms do not control for false positives. And false positive there are! Indeed, they found that most tractograms produced in this competition were made up of more invalid than valid bundles (52). Hence, they suggested using brain dissection for validation to avoid anatomy misrepresentation (16). These results instruct us to be cautious about dMRI data interpretation.

In terms of validation, *ex vivo* dMRI in cadaveric samples appears ideally suited to the study of normal anatomy as it can be obtained with maximal quality. There is no movement of artifacts, a very strong magnetic field can be used, and the scan can last many hours, which allows for a very small voxel size and high signal-to-noise ratio compared to clinical scans (53). A potential new method for validation was presented by Zemmoura et al. (54) Fibrascan is an approach designed by the authors allowing 3D white matter tract dissection in the cadaveric brain while using the *ex vivo* MR reference space to allow adequate correspondence.

#### What Diffusion Imaging Revealed About the SLF

Initial *in vivo* studies in non-human primates concluded that the SLF can structurally be divided into four independent components, the SLF I, SLF II, SLF III, and arcuate fasciculus (15). Yet, as the human brain is significantly different from other primates, especially in the peri-Sylvian areas, this nomenclature should obviously not be extrapolated to humans without confirmation (5, 55, 56).

Hence, the first step in human imaging studies was to survey for the presence of these 4 SLF subdivisions (15). Makris et al. successfully segmented the four SLF subcomponents in humans. In their nomenclature, the SLF I represents the dorsal division. It connects the superior parietal and superior frontal lobes. The SLF II takes its origin from the angular gyrus, passes through the core of the centrum semi-ovale above the insula, and ends in the caudal–lateral prefrontal region. The SLF III extends from the supramarginal gyrus, anterior to the angular gyrus, to the ventral premotor and prefrontal areas. SLF III is the most ventral of these three subdivisions. The fourth subcomponent of the SLF is homologous to the SLF IV described previously in non-human primates and corresponds to the AF. Its trajectory connects the caudal part of the superior temporal gyrus with the lateral prefrontal cortex, passing through the caudal end of the Sylvian fissure (15). Similar parcellation of the SLF that comprises the AF has also been described by subsequent investigators (57, 58). However, the inclusion of the AF as the 4th component of the SLF is far from unanimous and remains a subject of controversy in the nomenclature that is addressed in a later section of this study.

**Figure 2:**
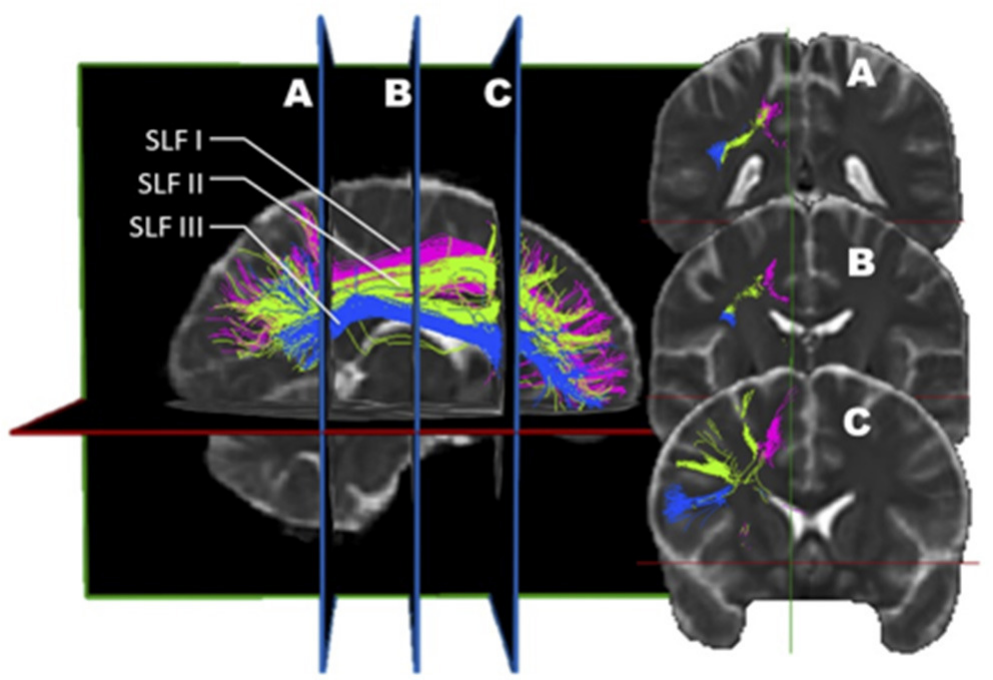
*In vivo* fiber tractography of the right SLF I (pink), SLF II (green), and SLF III (blue). A three-dimensional reconstruction of the three segments of the SLF is displayed at the left of the figure. The coronal sections of it (A, B, and C) are represented on the right. The dorso-ventral as well as medio-lateral localization of each sub-segment can be visualized on the coronal planes.

The first three SLF subdivisions are usually reported alike in most studies, except for a few minor differences (Figure 2). For example, Cabeen et al. (59) noted that the trajectory of the SLF I passes through the corona radiata and the superior lateral projections of the corpus callosum. Additionally, they found that the SLF II crosses the frontal lateral projections of the corpus callosum. It is as if, in this study, these two subdivisions were more dorsal than in the traditional description. In a distinctive description, Thiebaut de Schotten described the SLF as follows: the SLF I connects the precuneus, the superior parietal lobule, and Brodmann areas (BA) 5 and 7 to the superior frontal and anterior cingulate gyri, BA 8, 9, and 32. The SLF II originates at the angular gyrus and the anterior intraparietal sulcus, BA 39 and 40, and projects toward the posterior portions of the superior and middle frontal gyri, BA 8 and 9. The SLF III connects the temporoparietal junction, BA 40, with the inferior frontal gyrus, BA 44, 45, and 47, where Broca's area is localized (56). According to Schurr et al., SLF 2 and 3 would display different signatures when performing diffusion MRI. This discrepancy would be attributable to the fact that SLF 2 crosses the corona radiata fibers that run caudo-rostrally whereas SLF-3 obviously does not (60). Hence according to these authors, this anatomical divergence can be exploited to reliably identify SLF 2 and SLF3.

However, disparities in the description of this important fasciculus are not limited to these minor discrepancies. Indeed, some studies even describe a fifth division of the SLF comprised of a distinct fasciculus (61–63) that would connect the superior temporal gyrus to the superior parietal. This subdivision has been named “the temporoparietal SLF” (SLF TP) by Kamali (64). The SLF TP represents a distinct subcomponent of the SLF since it has a cranio-caudal orientation and is not in the longitudinal plane.

Furthermore, to emphasize how much this literature is still controversial, Bernal & Altman reported results calling into question much of the anatomical descriptions described above (57). Indeed, their study challenges the anatomical and functional foundation of the SLF. In this diffusion tensor imaging study, the SLF of 12 normal right-handed participants was assessed to find that projection to Broca's area was minimal or absent in the majority of cases. The only rostral endpoint of the SLF in this study was in the precentral gyrus. This finding contradicts the preconceived SLF structure and role, and does not represent the dominant consensus.

The high variability in the definition of the different divisions of the SLF between studies can probably be explained by a number of factors: the high individual variability combined with a limited number of participants accrued in the studies (65–68), as well as extreme variability in tractography algorithms and segmentation technique, leading to many false positives, are probably the major limitations (52). Recently, Schilling et al. studied the variability in results obtained by the segmentation of up to 14 bundles by 42 different groups (69). Even when given the same set of underlying streamlines, variability across protocols for bundle segmentation was found to be greater than all other sources of potential unevenness. The authors concluded that this extreme variance arose because of the poor inter-protocol agreements for the segmentation of many pathways. This is an illustration of the poor consensus on the precise anatomical definition of white matter bundles.

**Figure 3:**
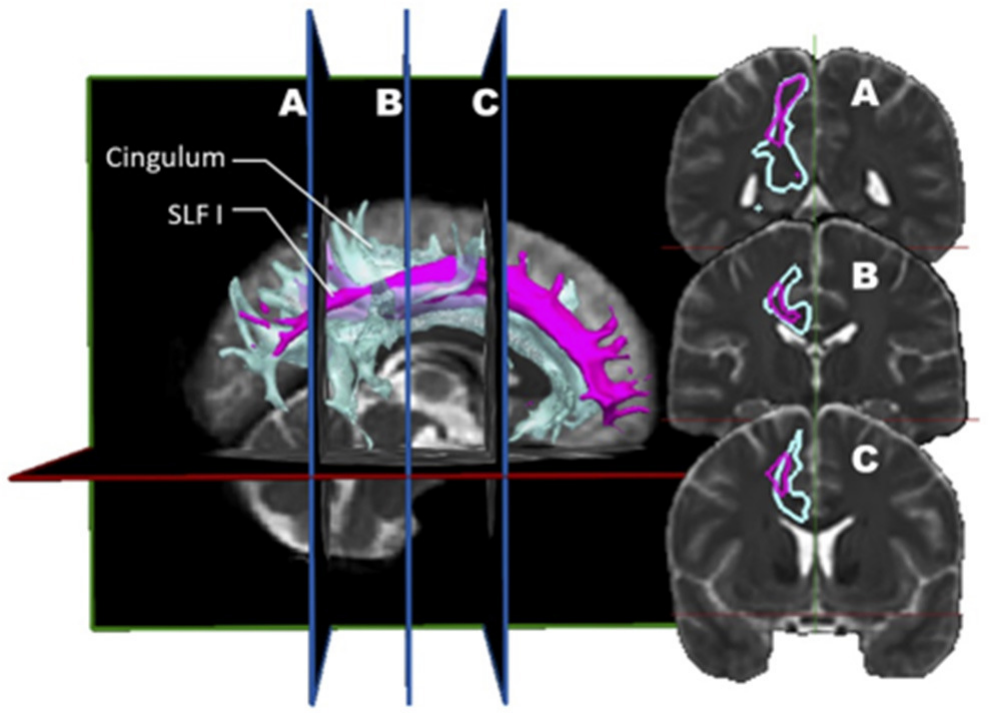
*In vivo* fiber tractography of the right SLF I (pink) and the right cingulum (light blue) represented by volume. A three-dimensional reconstruction of the SLF I and the cingulum is displayed at the left of the figure. The coronal sections of it (A, B and C) are represented on the right. On the coronal planes, it is possible to appreciate the great proximity between the fibers of the SLF I and those of the cingulum.

Ultimately, a study combining dMRI and a validation approach such as dissection would be ideal. Wang et al. (7) designed such a study. Before this report, few studies comparing diffusion imaging and postmortem dissection of the SLF had been published. Integrating both dMRI datasets (*n* = 10) and anatomical dissections (*n* = 5), this group analyzed the trajectory, volume, length, asymmetry, and cortical connectivity of the SLF in normal human brains. Their findings are rather provocative, differing from the rest of the literature and challenging the current knowledge on the SLF. The first conclusion is that there is a high discrepancy between the simian and human anatomy; hence, all conclusions derived from the study of the former cannot be applied to the latter. The second conclusion is more disturbing as it questions the actual nomenclature. In their opinion, the SLF I should no longer be considered as it depicts a spatial relationship so close to the cingulum to be indistinguishable from it (Figure 3). The cingulum is described in the literature as being the communication bundle between different regions of the limbic system (7). It forms the main component of the white matter corpus of the cingulate gyrus, running around the medial aspect of the frontal and parietal lobe, just above the corpus callosum. It projects its cingulate gyrus afferences to the entorhinal cortex of the temporal lobe. In a very close anatomical relationship, the SLF1 connects the superior frontal gyrus to the precuneus. Hence, they consider the SLF as only composed of a dorsal (SLF II) and a ventral (SLF III) segment. In this construct, the SLF II represents 66% of the total volume, whereas the SLF III about 33%. Although the SLF was present in both hemispheres, a distinct asymmetry was also observed. This asymmetric pattern affects mostly the SLF II. In the left hemisphere, the connectivity pattern was in keeping with a predominant functional role in speech (connections between the supramarginal gyrus with the dorsal precentral gyrus and caudal middle frontal gyrus). Interestingly, these frontal areas also receive connections from the AF. Hence, this connectivity profile supports the notion that the left SLF II (dorsal) is involved in the motor planning of language and/or syntactic processing during language production, and its role is distinct from the AF.

Interestingly, the connectivity pattern identified on the right was divergent, with the SLF II connecting the angular gyrus and the superior parietal lobe with the caudal and rostral middle frontal gyrus. This anatomical scheme, according to the authors, was congruent with a role in regulating the focus of attention in a spatial orientation. The main contribution of cadaveric dissections in this study was the characterization of the proximity between SLF I, cingulum, and AF, explaining the frequent confusion in the definition of these bundles in prior studies, according to the authors. Finally, when discussing the potential biases and limitations in their study, the authors highlighted the low number of subjects that could not balance the inter-individual variability.

Other authors also support the idea that the SLF is indeed comprised of two main divisions. Martino et al. used dMRI and cortex-sparing fiber dissection and came to the conclusion that there were two segments to the SLF, an anterior and a posterior segment, much like Martino et al. and Wang et al. (3, 7).

A summary of dissection and imaging studies relative to the characteristics of the SLF subdivisions is presented in Table 1.

**Table 1:** A summary of dissection and imaging studies relative to the characteristics of the SLF subdivisions.

**Number of SLF divisions**	**Studies**	**Technique used**	**Name of the bundle**	**Origin**	**Projection**	**Potential function**
2 or 2 and the AF	Zhang et al. (63)	dMRI	Anterior segment	Medial and inferior frontal lobe	Angular and supramarginal gyri	-
			Posterior segment	Angular and supramarginal gyri	Temporal lobe	-
			Long segment	Medial and inferior frontal lobe	Temporal lobe	-
	Martino et al. (16)	dMRI and dissections	Horizontal segment of the SLF	Inferior parietal lobe and posterior portion of the superior temporal gyrus	Frontal operculum	-
			Vertical segment of the SLF	Posterior portion of the middle temporal gyrus	Angular gyrus	-
			AF	Middle and inferior temporal gyrus	Posterior portion of the frontal operculum	-
	Martino et al. (3)	dMRI and dissections	Anterior segment	Supramarginal and superior temporal gyri	Precentral gyrus	Monitoring of speech articulation
			Posterior segment	Posterior portion of the middle temporal gyrus	Angular gyrus	Language perception (syllabe discrimination and identification)
			AF (long segment)	Middle and inferior temporal gyri	Precentral gyrus and posterior portion of the inferior and middle frontal gyri	Language function
	Zemmoura et al. (54)	dMRI and dissections	Anterior horizontal segment	Ventral premotor cortex	Inferior parietal lobule	-
			Posterior vertical segment	Inferior parietal lobule	Posterior superior and middle temporal gyri	-
			Long segment or AF	Pars opercularis and pars triangularis of the inferior frontal gyrus	Posterior middle temporal gyrus	-
	de Benedictis et al. (58)	dMRI and dissections	SLF II (Anterior component of the indirect component of the SLF)	Inferior frontal gyrus (Broca ‘s territory)	Inferior parietal lobule	-
			SLF III (Posterior component of the indirect component of the SLF)	Inferior parietal lobule	Posterior part of the superior and middle temporal gyrus (Wernicke ‘s territory)	-
			AF (Direct component of the SLF)	Inferior frontal gyrus (Broca ‘s territory)	Posterior part of the superior and middle temporal gyrus (Wernicke ‘s territory)	-
	Wang et al. (7)	dMRI and dissections	Dorsal segment (SLF II) in the left hemisphere	Angular gyrus, Brodmann Areas (BA) 39	Caudal middle frontal gyrus and dorsal precentral gyrus	Motor planning of language function and/or syntactic processing during language production
			Dorsal segment (SLF II) in the right hemisphere	Angular gyrus and the superior parietal lobe	Caudal and rostral middle frontal gyrus	regulating the focusing of attention in spatial orientation
			Ventral segment (SLF III) in the left hemisphere	Supramarginal gyrus (BA 40)	Ventral precentral gyrus and pars opercularis	Language function
			Ventral segment (SLF III) in the right hemisphere	Supramarginal gyrus (BA 40)	Pars triangularis	Spatial awareness
3	De Schotten et al. (9)	dMRI	SLF I	-	-	-
			SLF II	-	-	Visuospatial for the right SLF II
			SLF III	-	-	-
	Catani and Thiebaut de Schotten (70)	dMRI	SLF I	Parietal precuneus	Medial and superior surface of the superior frontal gyrus	Processes the spatial coordinates of trunk and inferior limbs, movement planning, oculomotor coordination and visual reaching
			SLF II	Posterior region of the inferior parietal lobule	Lateral aspect of the superior and middle frontal gyrus	Processes the spatial coordinates of upper limbs and other functions similar to the SLF I
			SLF III	Supramarginal and anterior angular gyrus	Posterior region of the inferior frontal gyrus	Sensory-motor function or language function
	Thiebaut de Schotten et al. (56) and Lunven and Bartolomeo (71)	dMRI	SLF I	Superior parietal lobule and precuneus (BA 5, 7)	Superior frontal and anterior cingulate areas (BA 8, 9, 32)	-
			SLF II	Angular gyrus and the anterior intraparietal sulcus (BA 39, 40)	Posterior regions of the superior and middle frontal gyri (BA 6, 8, 9)	Visuospatial for the right SLF II
			SLF III	Intraparietal sulcus and inferior parietal lobule (BA 40)	Inferior frontal gyrus (BA 44, 45, 47)	-
	Hecht et al. (55)	dMRI	SLF I	Superior parietal cortex	Superior frontal gyrus	Motor regulation
			SLF II	Posterior inferior parietal cortex	Middle frontal gyrus and dorsolateral prefrontal cortex	Overt and imagined mouvements, spatial orienting and spacial attention
			SLF III	Anterior inferior parietal cortex	Inferior frontal gyrus	Tool use and social learning mainly for the right SLF III
	Cabeen et al. (59)	dMRI	SLF I	Parietal cortex	Superior frontal gyrus	-
			SLF II		Middle frontal gyrus	-
			SLF III		Inferior frontal gyrus	-
	Schurr et al. (60)	dMRI	SLF I	-	-	-
			SLF II	-	-	-
			SLF III	Supramarginal gyrus and angular gyrus	Opecular and triangular parts of the inferior frontal gyrus for the left SLF and inferior frontal gyrus for the right SLF	-
3 and the AF	Makris et al. (15)	dMRI	SLF I	Superior parietal and superior frontal lobes	Dorsal premotor and dorsolateral prefrontal regions	Regulation of higher aspects of motor behavior
			SLF II	Angular gyrus	Caudal-lateral prefrontal regions	Perception of the visual space
			SLF III	Supramarginal gyrus	Ventral premotor and prefrontal regions	Articulatory component of langage and working memory
			AF	Caudal part of the superior temporal gyrus	Lateral prefrontal cortex	Receive and modulate audiospatial information
	Makris et al. (62)	dMRI	SLF I	-	-	-
			SLF II	-	Anterior part of the angular gyrus	-
			SLF III	-	-	-
			AF	-	Caudal part of the superior and middle temporal gyri	-
	Bernal and Altman (57)	dMRI	SLF I	Posterior temporoparietal area (Posterior langage areas)	Frontal areas (mainly in the precentral gyrus and minimally in Broca's area)	-
			SLF II			-
			SLF III			-
			AF	Temporal lobe		Involved in langage fonction, but not necessary for it.
5	Kamali et al. (64)	dMRI	SLF I	Superior parietal lobule along the cingulate gyri (BA 7, 5, 4)	Dorsal and medial cortex of the frontal lobe and premotor areas (BA 6, 8, 9)	Language
			SLF II	Angular gyrus (BA 39)	Passes through the post central gyrus (BA 3, 1, 2), the precentral gyrus (BA 4), the middle frontal gyrus (BA 6, 46) and terminates in the dorsolateral prefrontal cortex (BA 6, 8, 46)	
			SLF III	Supramarginal gyrus	Ventral premotor and prefrontal cortex (BA 6, 44)	
			AF	Posterior part of the superior temporal gyrus at the temporoparietal junction	Dorsal prefrontal cortex (BA 8, 46)	
			Temporoparietal SLF	Posterior part of the superior temporal gyrus at the temporoparietal junction	Angular gyrus and superior parietal lobule (BA 7)	

## Lateralization

We briefly touched on this aspect in the prior section with the study by Wang et al. But several other studies have investigated this with different conclusions. Let us first consider the non-controversial findings. If we are to still consider the SLF I as part of the SLF, then this trunk appears to be distributed symmetrically between the right and left hemispheres (9). The other finding has to do with the volume of the bundles; overall, the right-sided SLF as a whole appears more voluminous than on the left (3, 55). This is not consensual; however, as other researchers found the SLF II subcomponents' lateralization to be dominant in the left hemisphere (7, 56, 72). This would functionally be related to its role in language. Wang et al., on the other hand, found a slight asymmetric difference in all SLF sub-component with a tendency toward right-hemispheric dominance (7).

It stands to reason that there would be asymmetric findings between both SLF. As there is a clear asymmetry in speech function (left-sided characteristically) and visuospatial functioning (right-sided characteristically), some difference in connectivity is expected. Maybe SLF right and left are two completely different entities.

## Individual Variability

The inherent anatomical variability between an individual's brain cannot be emphasized enough. This variability can be observed at different levels such as in cortical morphology, cytoarchitecture, task-evoked activation, or in dMRI connectivity pattern (65–68). Hence, the idea of a prototypic universal brain blueprint is reductive. Although some areas and connectivity routes are highly conserved amongst individuals (such as primary areas and large projection bundles), others are more variable. This variability is a correlate of the phenotypic variability in general, determined by the genetic substrates and the environmental exposure that shapes this diversity for any trait, including the brain's anatomy, (68) and in fact, it goes even further than interindividual variability, as there is a clear intraindividual variability over time. Indeed, the brain connectivity pattern of an individual is constantly changing, and the density of white matter has been shown to change during the development of the individual. More so, later in life, the degradation of associative pathways due to lesions or neurodegenerative diseases is another cause of intraindividual variability (73).

With regard to the SLF specifically, there is diversity within the population. More precisely, from one individual to another, the volume of the SLF appears different. Fractional anisotropy of SLF also varies between individuals, but less importantly, than volume (67, 68). Hence, we need to apply a precautionary principle to the conclusions from all these anatomical studies, acknowledging the inherent variability from patient to patient. There is no such thing as a singular brain blueprint! Although these efforts to characterize the long associative fibers in the brain are paramount, it must be viewed with humility, considering that these long associative axons comprise only 2% of the total intrahemispheric cortico-cortical fibers and that 98% of the fibers are short U-fibers (74).

## Is The AF Part of the SLF?

Sometimes, various descriptions from different authors can give rise to confusion in anatomical classifications and subdivisions. Hence, clarifications in terms are in order. Such appears to be the case for the arcuate fasciculus (AF). As discussed earlier in this study, ever since the term Arcuate Fasciculus (AF) was incepted in the literature, confusion with the SLF was entertained.

However, if we use adequate definition in terms, some clarity emerges. Indeed, the SLF is in its essence a fronto-parietal tract, hence connecting the frontal and parietal cortices, whereas the AF is a fronto-temporal tract that passes through the parietal white matter beneath the SLF (55, 56). As such, the AF is recognized as a distinctive bundle with connection areas and a trajectory different from that of the SLF; if the 2 bundles are sometimes confused as synonyms, it is only because of an outdated nomenclature which should be abandoned. A great many studies clearly distinguish the 2 bundles as distinct entities, both anatomically and functionally (56, 59, 70, 71, 75, 76).

**Figure 4:**
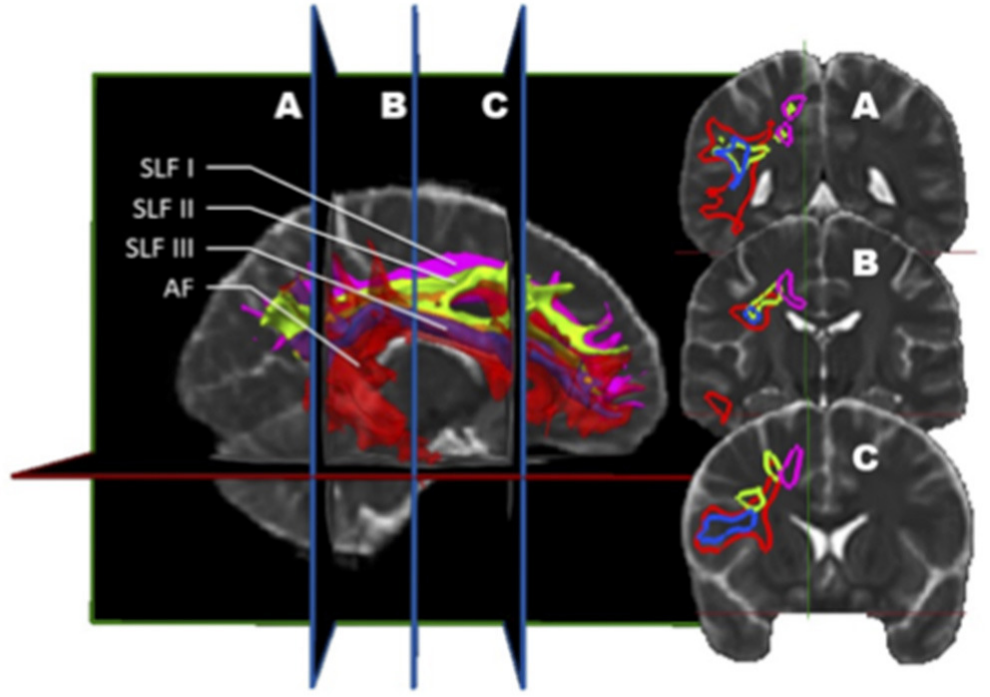
*In vivo* fiber tractography of the right SLF I (pink), SLF II (green), SLF III (blue), and AF (red) represented by volume. A three-dimensional reconstruction of these bundles is displayed at the left of the figure. The AF is slightly transparent to let appear the SLF III, being inside the latter. On the right, the coronal sections (A, B, and C) are represented. This figure confirms that the SLF III also corresponds to a sub-division of the AF. Indeed, throughout its length, the SLF III is located inside the AF.

However, this does not entirely solve the controversy. Surveying the literature on the AF with scrutiny, a detailed description emerges where this bundle can be divided into three segments (a long, anterior, and posterior segment), each connecting two regions of either Broca, Wernicke, or the territory of Geschwind (inferior parietal lobule). In this detailed description by Catani and Thiebaut de Schotten (70), the anterior segment of the AF appears to somehow correspond to the SLF III, and the two terms are used interchangeably. Hence, although these 2 bundles are distinct entities, some of their subcomponents appear to overlap as if they each share a subdivision: the SLF III and the anterior segment of the AF (Figure 4). This issue will require further insight from anatomico-functional studies.

## Nomenclature

Based on all that was discussed in this study, we feel compelled to recommend the adoption of a careful definition in the nomenclature of the SLF. There are clear and obvious reasons as to why the definition of the SLF remains controversial despite numerous studies. The persisting confusion regarding the distinction between the SLF and the AF up to this day is the epitome of this confusion. Moreover, a lot of the prior work which has made its way in the literature and anatomy textbooks is based on non-human primates, which present a rather different connectivity anatomy, thus steering the nomenclature toward a biased pattern (5, 56). Finally, as mentioned before, the individual variability had been underestimated in the past. The recognition of this fact foreshadows a future take on brain study connectivity that will key in this variability in study design and conclusions.

**Figure 5:**
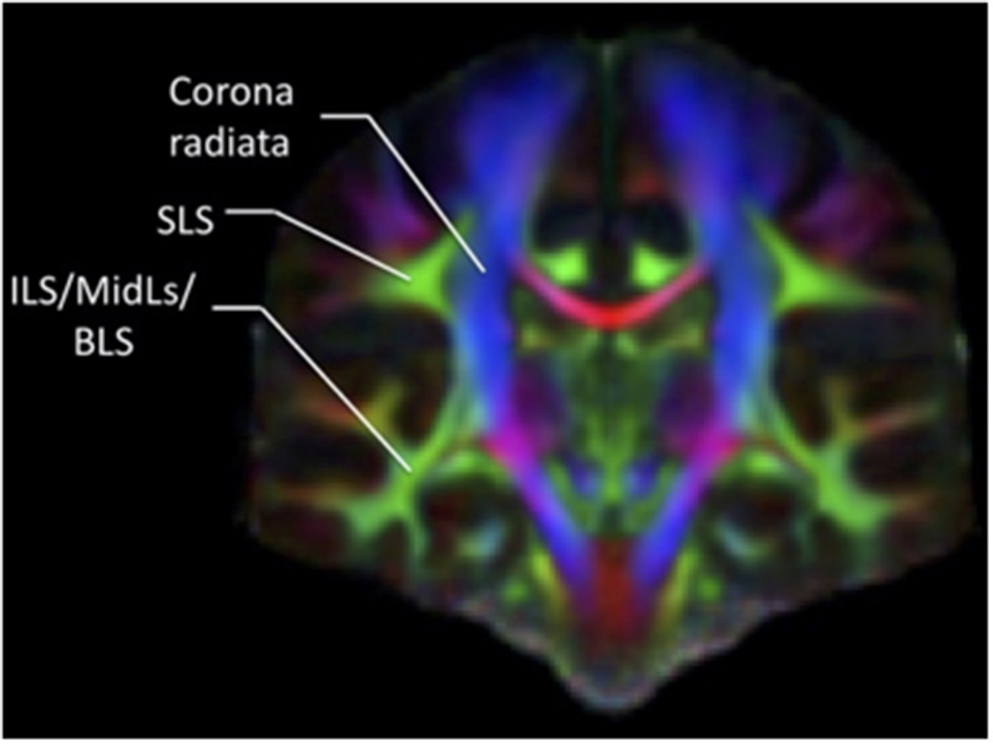
Coronal section of a color-coded FA map. The green associative fibers, representing the fibers perpendicular to the plane, can be grouped into two main systems according to the classification by Mandonnet et al. (5), the SLS and the common stem for the ILS/MidLs and BLS.

**Figure 6:**
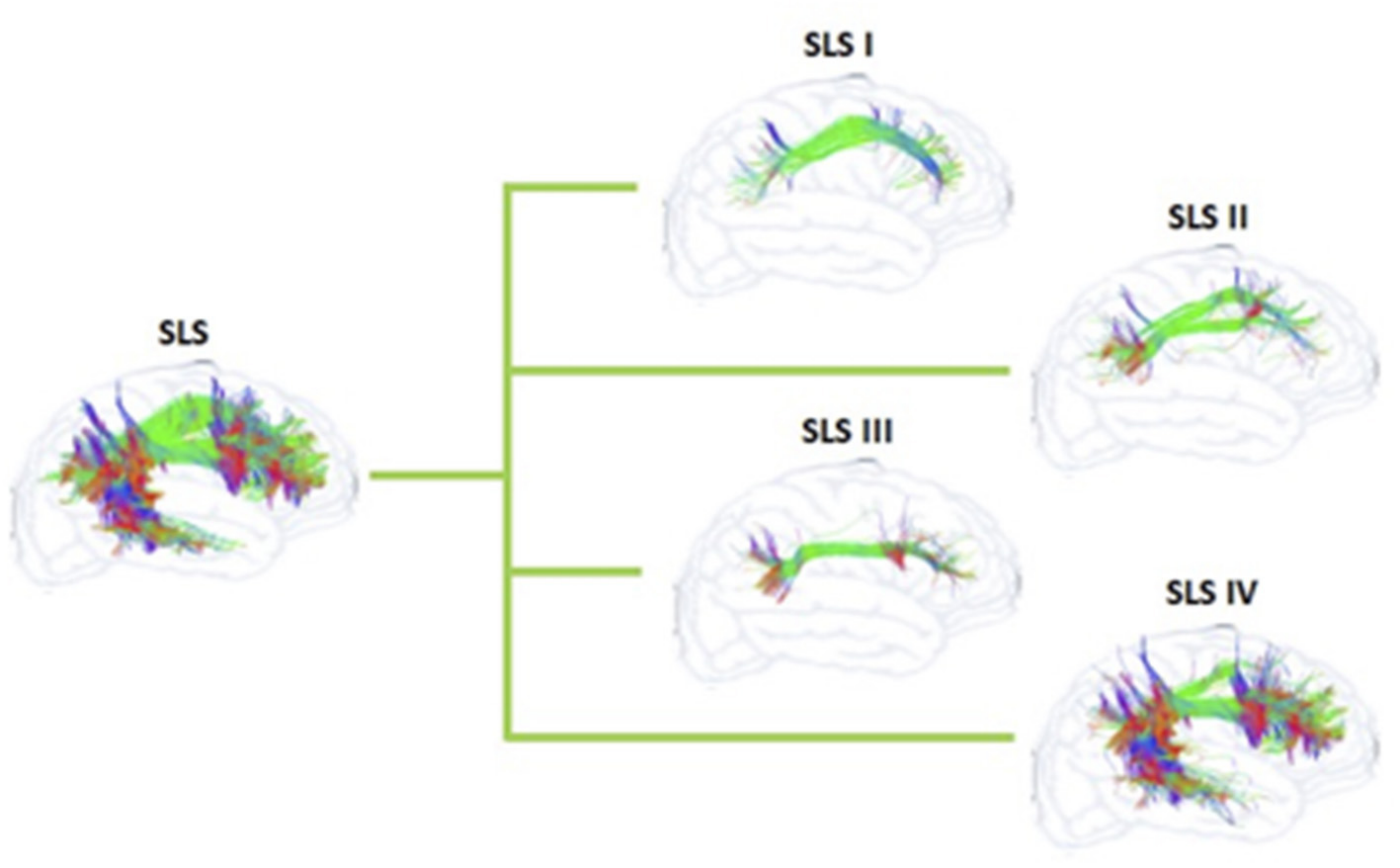
Schematization of the SLS and its sub-systems of the proposed nomenclature by Mandonnet et al. (5) for the human white matter association pathways.

The confusion regarding the actual nomenclature is such that some authors actually suggest erasing the historical connotations and starting from scratch. Recently, Mandonnet et al. (5) suggested the use of anatomical corridors available for the fanning of longitudinal fibers in the brain, in relation to brain landmarks (such as ventricles, grey nuclei, external/extreme capsule) to define a stem-based white-matter nomenclature (Figure 5). Hence, they propose seven white matter systems: the superior (SLS), inferior (ILS), and middle longitudinal system MidLS), the basal (BSL) and mesial longitudinal system (MesLS), and the anterior (ATS) and posterior transverse system (PTS). This classification is further complexified by a second level to more precisely identify the cortical areas connected by the fasciculi. As an example, the SLS and the ILS each have 5 divisions. In this scheme, SLS I is equivalent to SLF I, SLS II to SLF II, and SLS III to SLF III. The authors chose to voluntarily ignore historical references by erasing commonly used terms in the literature. Hence, the AF becomes the SLS IV (Figure 6), the uncinate fasciculus becomes the ILS IV, the cingulum the MesLS I, and so forth (5). The rationale of defining “anatomical corridors” as a correlate for naming white matter fibers' propagation is interesting. Now, whether this new classification will succeed in erasing centuries of historical references remains to be seen. However, this might be beneficial, allowing us to redefine better the longitudinal connection pathways in the human using data from recently designed studies, and burying old misconceptions still blurring our literature. Vavassori et al. adopt a similar approach, rewriting the superior longitudinal system in terms of wiring diagram describing the connecting cortices and following a medio-lateral distribution, instead of focusing on specific bundles (77). As an additional distinction, we would also urge investigators to consider the left and right hemispheres as distinct entities in their analyses.

## Functional Correlates of SLF

The SLF is a massive bundle connecting widespread areas of the frontal and the parietal lobes. As such, it is likely involved in different functional correlates. As the bundle depicts clear asymmetry between both hemispheres, this must translate into obvious functional implications. Hence, although still a controversial matter, the SLF III shares similar connectivity with the AF, linking the inferior frontal gyrus with the ventral pre-central gyrus. This suggests, in the left hemisphere, a role in speech. This view has been confirmed by Maldonado et al. (78) through a brain electrostimulation study. Wang et al. (7) also reported a specific connectivity pattern for the right SLF III terminating at the right pars triangularis. According to some authors, this connectivity pattern is probably involved in spatial awareness functioning (7).

A strong asymmetry also seems to characterize the SLF II. Wang et al. (7) identified a strong bias in leftward connections between the supramarginal gyrus with the dorsal precentral gyrus and the caudal middle frontal gyrus. This would be in keeping with a role for the left SLF II in motor planning of speech and/or syntax processing. In a similar predicament as for the SLF III, the right SLF II preferentially connects the angular gyrus and the superior parietal lobe with the caudal and rostral middle frontal gyrus, respectively. It is presumed that this system plays a role in the regulation of the attention in a spatial orientation. This is in keeping with the fact that the right SLF II is responsible for a faster and preferential visuospatial processing in the right hemisphere (9).

## Conclusion

“There is no such thing as absolute certainty, but there is assurance sufficient for the purposes of human life” as once said by John Stuart Mill. Through this review and analysis of data regarding the state-of-the-art knowledge on the anatomical and functional correlates of the SLF, we can come to the conclusion that there is only one certainty: we do not know for sure!

Indeed, the SLF itself in its current definition is not a consensual entity. However, we can stand up on some of the groundwork cited in this article, and presumably conclude the following: (1) that the very definition of the SLF, connecting the frontal to the parietal lobe, allows a clear distinction with the AF, connecting the frontal lobe to the temporal lobe. Hence, all classifications including the AF as part of the SLF are misleading. (2) Traditionally, the SLF has been divided into 3 segments. However, the SLF I connecting the parietal precuneus to the superior frontal gyrus appears assimilable to the cingulum. Hence, its existence, as well as its role is unclear at this time. (3) The SLF II and III are clearly defined as distinct entities that are asymmetrical in their function as well as presumably in their role: the left-sided SLF II and III would definitely be involved in speech processing, whereas the right-sided SLF II and III would contribute to visuospatial functioning. (4) The inter as well as intra-individual variability in connectivity is more important than previously suspected. Hence, we need to apply a precautionary principle to the conclusions from the connectivity studies, acknowledging the inherent variability from patient to patient. Mitigation of this limitation can be achieved through the use of larger study cohorts.

## Author's Note

All figures were created using open-source MRI images as well as the whole brain tractogram of the test dataset of Imeka (79). The detailed methodology for Cingulum segmentation was derived from Wakana et al. (80). The detailed methodology for SLF I, II, and III segmentation, as well as AF segmentation, was emulated from Fitzgerald et al. (81).

## Author Contributions

FJ, CI-M, and DF were responsible for the design of the manuscript. FJ did the literature search. CI-M and DF reviewed and interpreted the data and were responsible for the analysis. DF completed the final version of the manuscript and recommendations about suggested nomenclature. All authors contributed to the article and approved the submitted version.

## Funding

This work was financed by La fondation coeur en tête on brain tumor research.

## Conflict of Interest

The authors declare that the research was conducted in the absence of any commercial or financial relationships that could be construed as a potential conflict of interest.

## Publisher's Note

All claims expressed in this article are solely those of the authors and do not necessarily represent those of their affiliated organizations, or those of the publisher, the editors and the reviewers. Any product that may be evaluated in this article, or claim that may be made by its manufacturer, is not guaranteed or endorsed by the publisher.

## References

[1] Honey CJ Kötter R Breakspear M Sporns O. Network structure of cerebral cortex shapes functional connectivity on multiple time scales. PNAS. (2007) 104:10240–5. 10.1073/pnas.0701519104 17548818 PMC1891224

[2] de Benedictis A Duffau H. Brain hodotopy: from esoteric concept to practical surgical applications. Neurosurgery. (2011) 68:1709–23. 10.1227/NEU.0b013e3182124690 21346655

[3] Martino J De Witt Hamer P Berger M T Lawton M Arnold C. Analysis of the subcomponents and cortical terminations of the perisylvian superior longitudinal fasciculus: a fiber dissection and DTI tractography study. Brain Struct Funct. (2013) 218:105–21. 10.1007/s00429-012-0386-5 22422148

[4] Catani M Dell'acqua F Bizzi A Forkel S Williams S Simmons A. Beyond cortical localization in clinico-anatomical correlation. Cortex. (2012) 48:1262–87. 10.1016/j.cortex.2012.07.001 22995574

[5] Mandonnet E Sarubbo S Petit L. The nomenclature of human white matter association pathways: proposal for a systematic taxonomic anatomical classification. Front Neuroanat. (2018) 12:1–14. 10.3389/fnana.2018.00094 30459566 PMC6232419

[6] Petrides M Pandya DN. Projections to the frontal cortex from the posterior parietal region in the rhesus monkey. J Comparat Neurol. (1984) 228:105–16. 10.1002/cne.902280110 6480903

[7] Wang X Pathak S Stefaneanu L Yeh F-C Li S Fernandez-Miranda JC. Subcomponents and connectivity of the superior longitudinal fasciculus in the human brain. Brain Struct Funct. (2016) 221:2075–92. 10.1007/s00429-015-1028-5 25782434

[8] Pandya DN. Kuypers HG. Cortico-cortical connections in the rhesus monkey. Brain Res. (1969) 13:13–36. 10.1016/0006-8993(69)90141-3 4185124

[9] Thiebaut de Schotten M Dell'Acqua F Forkel SJ Simmons A Vergani F Murphy DGM. A lateralized brain network for visuospatial attention. Nat Neurosci. (2011) 14:1245–6. 10.1038/nn.2905 21926985

[10] Kawamura K Naito J. Corticocortical projections to the prefrontal cortex in the rhesus monkey investigated with horseradish peroxidase techniques. Neurosci Res. (1984) 1:89–103. 10.1016/S0168-0102(84)80007-3 6536892

[11] Basser PJ Pajevic S Pierpaoli C Duda J Aldroubi A. In vivo fiber tractography using DT-MRI data. Magnetic Resonance in Medicine. (2000) 44:625–32. 10.1002/1522-2594(200010)44:4<625::AID-MRM17>3.0.CO;2-O 11025519

[12] Mori S Crain BJ Chacko VP van Zijl PC. Three-dimensional tracking of axonal projections in the brain by magnetic resonance imaging. Ann Neurol. (1999) 45:265–9. 10.1002/1531-8249(199902)45:2&lt;265::AID-ANA21&gt;3.0.CO;2-3 9989633

[13] Mukherjee P Berman JI Chung SW Hess CP Henry RG. Diffusion tensor MR imaging and fiber tractography: theoretic underpinnings. AJNR. (2008) 29:632–41. 10.3174/ajnr.A1051 18339720 PMC7978191

[14] Archip N Clatz O Whalen S Kacher D Fedorov A Kot A. Non-rigid alignment of pre-operative MRI, fMRI, and DT-MRI with intra-operative MRI for enhanced visualization and navigation in image-guided neurosurgery. Neuroimage. (2007) 35:609–24. 10.1016/j.neuroimage.2006.11.060 17289403 PMC3358788

[15] Makris N Kennedy DN McInerney S Sorensen AG Wang R Caviness Jr VS. Segmentation of subcomponents within the superior longitudinal fascicle in humans: a quantitative, in vivo, DT-MRI study. Cereb Cortex. (2005) 15:854–69. 10.1093/cercor/bhh186 15590909

[16] Martino J De Witt Hamer PC Vergani F Brogna C de Lucas EM Vázquez-Barquero A. Cortex-sparing fiber dissection: an improved method for the study of white matter anatomy in the human brain. J Anat. (2011) 219:531–41. 10.1111/j.1469-7580.2011.01414.x 21767263 PMC3196758

[17] Maldonado IL Mandonnet E Duffau H. Dorsal fronto-parietal connections of the human brain: a fiber dissection study of their composition and anatomical relationships. Anat Rec. (2012) 295:187–95. 10.1002/ar.22462 22190345

[18] Chu W-S Furusato B Wong K Sesterhenn IA Mostofi FK Wei MQ. Ultrasound-accelerated formalin fixation of tissue improves morphology, antigen and mRNA preservation. Mod Pathol. (2005) 18:850–63. 10.1038/modpathol.3800354 15605077

[19] Schmierer K Kingshott CAMW Tozer DJ Boulby PA Parkes HG Yousry TA. Quantitative magnetic resonance of postmortem multiple sclerosis brain before and after fixation. Magnetic Resonance in Medicine. (2008) 59:268–77. 10.1002/mrm.21487 18228601 PMC2241759

[20] Stüber C Morawski M Schäfer A Labadie C Wähnert M Leuze C. Myelin and iron concentration in the human brain: a quantitative study of MRI contrast. NeuroImage. (2014) 93:95–106. 10.1016/j.neuroimage.2014.02.026 24607447

[21] van Duijn S Nabuurs RJA van Rooden S Maat-Schieman MLC van Duinen SG van Buchem MA. artifacts in human brain tissue after prolonged formalin storage. Magnetic Resonance in Medicine. (2011) 65:1750–8. 10.1002/mrm.22758 21305598

[22] Makris N Meyer JW Bates JF Yeterian EH Kennedy DN Caviness VS. MRI-Based topographic parcellation of human cerebral white matter and nuclei II. Rationale and applications with systematics of cerebral connectivity. NeuroImage. (1999) 9:18–45. 10.1006/nimg.1998.0384 9918726

[23] Caviness VS Meyer JW Makris N Bates JF Yeterian EH Kennedy DN. MRI-based topographic parcellation of human cerebral white matter and nuclei. Neuroimage. (2002). 9918725 10.1006/nimg.1998.0383

[24] Hagmann P Jonasson L Maeder P Thiran JP Wedeen VJ Meuli R. Understanding diffusion MR imaging techniques: from scalar diffusion-weighted imaging to diffusion tensor imaging and beyond. Radiographics. (2006) 26:S205–S223. 10.1148/rg.26si065510 17050517

[25] Alexander DC Dyrby TB Nilsson M Zhang H. Imaging brain microstructure with diffusion MRI: practicality and applications. NMR Biomed. (2019) 32:e3841. 10.1002/nbm.3841 29193413

[26] Beaulieu C. The basis of anisotropic water diffusion in the nervous system - a technical review. NMR Biomed. (2002) 15:435–55. 10.1002/nbm.782 12489094

[27] Leclercq D Delmaire C de Champfleur NM Menjot de Champfleur N Chiras J Lehéricy S. Diffusion tractography: methods, validation and applications in patients with neurosurgical lesions. Neurosurg Clin N Am. (2011) 22:253–68. 10.1016/j.nec.2010.11.004 21435575

[28] Pierpaoli C Jezzard P Basser PJ Barnett A Di Chiro G. Diffusion tensor MR imaging of the human brain. Radiology. (1996) 201:637–48. 10.1148/radiology.201.3.8939209 8939209

[29] Stejskal EO Tanner JE. Spin diffusion measurements: spin echoes in the presence of a time-dependent field gradient. J Chem Phys. (2004) 42:288–92. 10.1063/1.1695690

[30] Alexander AL Lee JE Lazar M Field AS. Diffusion tensor imaging of the brain. Neurotherapeutics. (2007). 10.1016/j.nurt.2007.05.011 17599699 PMC2041910

[31] Abhinav K Yeh F-C Mansouri A Zadeh G Fernandez-Miranda JC. High-definition fiber tractography for the evaluation of perilesional white matter tracts in high-grade glioma surgery. Neuro Oncol. (2015) 17:1199–209. 10.1093/neuonc/nov113 26117712 PMC4588761

[32] Fernandez-Miranda JC. Editorial: Beyond diffusion tensor imaging. J Neurosurg. (2013) 118:1363–5. 10.3171/2012.10.JNS121800 23540267

[33] Descoteaux M. High Angular Resolution Diffusion Imaging (HARDI). Wiley Encyclopedia of Electrical and Electronics Engineering (EEEE). (2015).

[34] McDonald CR White NS Farid N Lai G Kuperman JM Bartsch H. Recovery of white matter tracts in regions of peritumoral FLAIR hyperintensity with use of restriction spectrum imaging. American Journal of Neuroradiology. (2013) 34:1157–63. 10.3174/ajnr.A3372 23275591 PMC3928241

[35] Wedeen VJ Hagmann P Tseng WYI Reese TG Weisskoff RM. Mapping complex tissue architecture with diffusion spectrum magnetic resonance imaging. Magn Reson Med. (2005) 54:1377–86. 10.1002/mrm.20642 16247738

[36] Wedeen VJ Wang RP Schmahmann JD Benner T Tseng WYI Dai G Pandya DN. Diffusion spectrum magnetic resonance imaging (DSI) tractography of crossing fibers. Neuroimage. (2008) 41:1267–77. 10.1016/j.neuroimage.2008.03.036 18495497

[37] Glenn GR Helpern JA Tabesh A Jensen JH. Quantitative assessment of diffusional kurtosis anisotropy. NMR Biomed. (2015) 28:448–59. 10.1002/nbm.3271 25728763 PMC4378654

[38] Hui ES Russell Glenn G Helpern JA Jensen JH. Kurtosis analysis of neural diffusion organization. Neuroimage. (2015) 106:391–403. 10.1016/j.neuroimage.2014.11.015 25463453 PMC4389769

[39] Jensen JH Helpern JA Ramani A Lu H Kaczynski K. Diffusional kurtosis imaging: The quantification of non-Gaussian water diffusion by means of magnetic resonance imaging. Magn Reson Med. (2005) 53:1432–40. 10.1002/mrm.20508 15906300

[40] Descoteaux M Angelino E Fitzgibbons S Deriche R. Regularized, fast, and robust analytical Q-ball imaging. Magn Reson Med. (2007) 58:497–510. 10.1002/mrm.21277 17763358

[41] Tuch DS. Q-ball imaging. Magn Reson Med. (2004) 52:1358–72. 10.1002/mrm.20279 15562495

[42] Jbabdi S Johansen-Berg H. Tractography: where do we go from here? Brain Connect. (2011). 10.1089/brain.2011.0033 22433046 PMC3677805

[43] Jeurissen B Leemans A Tournier JD Jones DK Sijbers J. Investigating the prevalence of complex fiber configurations in white matter tissue with diffusion magnetic resonance imaging. Hum Brain Mapp. (2013). 10.1002/hbm.22099 22611035 PMC6870534

[44] Dauguet J Peled S Berezovskii V Delzescaux T Warfield SK Born R. Comparison of fiber tracts derived from in-vivo DTI tractography with 3D histological neural tract tracer reconstruction on a macaque brain. Neuroimage. (2007) 37:530–8. 10.1016/j.neuroimage.2007.04.067 17604650

[45] Kaufman JA Ahrens ET Laidlaw DH Zhang S Allman JM. Anatomical analysis of an aye-aye brain (Daubentonia madagascariensis, primates: prosimii) combining histology, structural magnetic resonance imaging, and diffusion-tensor imaging. Anat Rec A Discov Mol Cell Evol Biol. (2005) 1026–37. 10.1002/ar.a.20264 16211637

[46] Fernandez-Miranda JC Rhoton AL Álvarez-Linera J Kakizawa Y Choi C De Oliveira EP. Three-dimensional microsurgical and tractographic anatomy of the white matter of the human brain. Neurosurgery. (2008) 62. 10.1227/01.neu.0000333767.05328.49 18695585

[47] de Benedictis A Petit L Descoteaux M Marras CE Barbareschi M Corsini F. New insights in the homotopic and heterotopic connectivity of the frontal portion of the human corpus callosum revealed by microdissection and diffusion tractography. Hum Brain Mapp. (2016) 37:4718–35. 10.1002/hbm.23339 27500966 PMC6867471

[48] Fernandez-Miranda JC Pathak S Engh J Jarbo K Verstynen T Yeh F. High-definition fiber tractography of the human brain: Neuroanatomical validation and neurosurgical applications. Neurosurgery. (2012) 71:430–53. 10.1227/NEU.0b013e3182592faa 22513841

[49] Dyrby TB Søgaard LV Parker GJ Alexander DC Lind NM Baaré WFC. Validation of in vitro probabilistic tractography. Neuroimage. (2007) 37:1267–77. 10.1016/j.neuroimage.2007.06.022 17706434

[50] Seehaus A Roebroeck A Bastiani M Fonseca L Bratzke H Lori N. Histological validation of high-resolution DTI in human post mortem tissue. Front Neuroanat. (2015) 9. 10.3389/fnana.2015.00098 26257612 PMC4511840

[51] Vanderweyen DC Theaud G Sidhu J Rheault F Sarubbo S Descoteaux M. The role of diffusion tractography in refining glial tumor resection. Brain Struct Funct. (2020) 34:E1–E24. 10.1007/s00429-020-02056-z 32180019

[52] Maier-Hein KH Neher PF Houde J-C Côté MA Garyfallidis E Chamberland M et al. The challenge of mapping the human connectome based on diffusion tractography. Nat Commun. (2017). 29116093 10.1038/s41467-017-01285-x PMC5677006

[53] Takahashi E Song JW Folkerth RD Grant PE Schmahmann JD. Detection of postmortem human cerebellar cortex and white matter pathways using high angular resolution diffusion tractography: a feasibility study. Neuroimage. (2013) 68:105–11. 10.1016/j.neuroimage.2012.11.042 23238434 PMC4393953

[54] Zemmoura I Serres B Andersson F Barantin F Tauber C Filipiak I Cottier JP Venturini G Destrieux C. FIBRASCAN: a novel method for 3D white matter tract reconstruction in MR space from cadaveric dissection. NeuroImage. (2014) 103:106–18. 10.1016/j.neuroimage.2014.09.016 25234114

[55] Hecht EE Gutman DA Bradley BA Preuss TM Stout D. Virtual dissection and comparative connectivity of the superior longitudinal fasciculus in chimpanzees and humans. Neuroimage. (2015). 10.1016/j.neuroimage.2014.12.039 25534109 PMC4324003

[56] Thiebaut de Schotten M Dell'acqua F Valabregue R Catani M. Monkey to human comparative anatomy of the frontal lobe association tracts. Cortex. (2011). 10.1016/j.cortex.2011.10.001 22088488

[57] Bernal B Altman N. The connectivity of the superior longitudinal fasciculus: A tractography DTI study. Magn Reson Imaging. (2010) 28:217–25. 10.1016/j.mri.2009.07.008 19695825

[58] de Benedictis A Duffau H Paradiso B Grandi E Balbi S Granieri E. Anatomo-functional study of the temporo-parieto-occipital region: Dissection, tractographic and brain mapping evidence from a neurosurgical perspective. J Anat. (2014). 10.1111/joa.12204 24975421 PMC4111924

[59] Cabeen RP Bastin ME Laidlaw DH. Kernel regression estimation of fiber orientation mixtures in diffusion MRI. Neuroimage. (2016). 10.1016/j.neuroimage.2015.11.061 26691524 PMC4870009

[60] Schurr R Zelman A Mezer A. A subdividing the superior longitudinal fasciculus using local quantitative MRI. NeuroImage. (2019) 116439 10.1016/j.neuroimage.2019.116439 31821870

[61] Frey S Campbell JSW Pike GB Petrides M. Dissociating the human language pathways with high angular resolution diffusion fiber tractography. J Neurosci. (2008) 28:11435–44. 10.1523/JNEUROSCI.2388-08.2008 18987180 PMC6671318

[62] Makris N Papadimitriou GM Kaiser JR Sorg S Kennedy DN Pandya DN. Delineation of the middle longitudinal fascicle in humans: A quantitative, in vivo, DT-MRI study. Cerebral Cortex. (2009). 10.1093/cercor/bhn124 18669591 PMC2651473

[63] Zhang Y Zhang J Oishi K. Atlas-guided tract reconstruction for automated and comprehensive examination of the white matter anatomy. Neuroimage. (2010) 52:1289–301. 10.1016/j.neuroimage.2010.05.049 20570617 PMC2910162

[64] Kamali A Flanders AE Brody J Hunter JV Hasan KM. Tracing superior longitudinal fasciculus connectivity in the human brain using high resolution diffusion tensor tractography. Brain Struct Funct. (2014). 10.1007/s00429-012-0498-y 23288254 PMC3633629

[65] Amunts K Weiss PH Mohlberg H Faria AV Jiang H Li X. Analysis of neural mechanisms underlying verbal fluency in cytoarchitectonically defined stereotaxic space - The roles of Brodmann areas 44 and 45. Neuroimage. (2004) 22:42–56. 10.1016/j.neuroimage.2003.12.031 15109996

[66] Baldassarre A Lewis CM Committeri G Snyder AZ Romani GL Corbetta M. Individual variability in functional connectivity predicts performance of a perceptual task. Proc Natl Acad Sci U S A. (2012) 3516–21. 10.1073/pnas.1113148109 22315406 PMC3295318

[67] Good CD Johnsrude IS Ashburner J Henson RN Friston KJ Frackowiak RS. voxel-based morphometric study of ageing in 465 normal adult human brains. Neuroimage. (2001). 10.1006/nimg.2001.0786 11525331

[68] Hill J Inder T Neil J Dierker D Harwell J Van Essen D. Similar patterns of cortical expansion during human development and evolution. PNAS. (2010) 107:13135–40. 10.1073/pnas.1001229107 20624964 PMC2919958

[69] Schilling KG Rheault F Petit L Hansen CB Nath V Yeh FC. Tractography dissection variability: what happens when 42 groups dissect 14 white matter bundles on the same dataset? Neuroimage. (2021) 243:118502. 10.1016/j.neuroimage.2021.118502 34433094 PMC8855321

[70] Catani M Thiebaut de Schotten M. Atlas of Human Brain Connections. Oxford: University Press. (2012). 10.1093/med/9780199541164.001.0001

[71] Lunven M Bartolomeo P. Attention and spatial cognition: neural and anatomical substrates of visual neglect. Ann Phys Rehabil Med. (2017) 60:124–9. 10.1016/j.rehab.2016.01.004 26874577

[72] Vernooij MW Smits M Wielopolski PA Houston GC Krestin GP van der Lugt A. Fiber density asymmetry of the arcuate fasciculus in relation to functional hemispheric language lateralization in both right- and left-handed healthy subjects: a combined fMRI and DTI study. Neuroimage. (2007) 35:1064–76. 10.1016/j.neuroimage.2006.12.041 17320414

[73] MacDonald SWS Nyberg L Bäckman L. Intra-individual variability in behavior: links to brain structure, neurotransmission and neuronal activity. Trends Neurosci. (2006) 29:474–80. 10.1016/j.tins.2006.06.011 16820224

[74] Duffau H. Brain Mapping, From Neural Basis of Cognition To Surgical Applications. Austria: Springer-Verlag/Wien. (2012).

[75] Thiebaut de Schotten M Ffytche DH Bizzi A Dell'Acqua F Allin M Walshe M. Atlasing location, asymmetry and inter-subject variability of white matter tracts in the human brain with MR diffusion tractography. NeuroImage. (2011) 54:49–59. 10.1016/j.neuroimage.2010.07.055 20682348

[76] Schmahmann JD Pandya DN. The complex history of the fronto-occipital fasciculus. J Hist Neurosci. (2007). 10.1093/acprof:oso/9780195104233.003.0019 17966054

[77] Vavassori L Sarubbo S Petit L. Hodology of the superior longitudinal system of the human brain: a historical perspective, the current controversies, and a proposal. Brain Struct Funct. (2021) 226:1363–84. 10.1007/s00429-021-02265-0 33881634

[78] Maldonado IL Moritz-Gasser S Duffau H. Does the left superior longitudinal fascicle subserve language semantics? A brain electrostimulation study. Brain Struct Funct. (2011) 216:263–74. 10.1007/s00429-011-0309-x 21538022

[79] Rheault FH Goyette JC Morency N Descoteaux FM. MI-Brain, a Software to Handle Tractograms and Perform Interactive Virtual Dissection. Lisbon: Breaking the Barriers of Diffusion MRI (ISMRM workshop) (2016).

[80] Wakana S Caprihan A Panzenboeck MM Fallon JH Perry M Gollub RL. Reproducibility of quantitative tractography methods applied to cerebral white matter. Neuroimage. (2007) 36:630–44. 10.1016/j.neuroimage.2007.02.049 17481925 PMC2350213

[81] Fitzgerald J Leemans A Kehoe E O'Hanlon E Gallagher L McGrath J. Abnormal fronto-parietal white matter organisation in the superior longitudinal fasciculus branches in autism spectrum disorders. Eur J Neurosci. (2018) 47:652–61. 10.1111/ejn.13655 28741714

